# Profiling the Proteome of Exhaled Breath Condensate in Healthy Smokers and COPD Patients by LC-MS/MS

**DOI:** 10.3390/ijms131113894

**Published:** 2012-10-29

**Authors:** Marco Fumagalli, Fabio Ferrari, Maurizio Luisetti, Jan Stolk, Pieter S. Hiemstra, Daniela Capuano, Simona Viglio, Laura Fregonese, Isa Cerveri, Federica Corana, Carmine Tinelli, Paolo Iadarola

**Affiliations:** 1 Department of Biology and Biotechnology, University of Pavia, Via Taramelli 3/B, 27100 Pavia, Italy; E-Mail: fummar07@unipv.it; 2 Lab Analysis Inc., Casanova Lonati, 27041 Pavia, Italy; E-Mail: f.ferrari@labanalysis.it; 3 Department of Molecular Medicine, Division of Pneumology, University of Pavia & IRCCS Policlinico San Matteo, Via Taramelli 5, 27100 Pavia, Italy; E-Mails: m.luisetti@smatteo.pv.it (M.L.); daniela.capuano83@gmail.com (D.C.); icerveri@smatteo.pv.it (I.C.); 4 Department of Pulmonology, Leiden University Medical Center, 2333 Leiden, The Netherlands; E-Mails: J.Stolk@lumc.nl (J.S.); p.s.hiemstra@lumc.nl (P.S.H.); laurafregonese@gmail.com (L.F.); 5 Department of Molecular Medicine, Division of Biochemistry, University of Pavia, Via Taramelli 3/B, 27100, Pavia, Italy; E-Mail: simona.viglio@unipv.it; 6 Centro Grandi Strumenti, University of Pavia, Via Bassi 6, 27100 Pavia, Italy; E-Mail: federica.corana@unipv.it; 7 Biometric Unit, IRCCS San Matteo Hospital Foundation, 27100 Pavia, Italy; E-Mail: ctinelli@smatteo.pv.it

**Keywords:** EBC, COPD, proteomics, LC-MS/MS

## Abstract

Three pools of exhaled breath condensate (EBC) from non-smokers plus healthy smokers (NS + HS, *n* = 45); chronic obstructive pulmonary disease (COPD) without emphysema (COPD, *n* = 15) and subjects with pulmonary emphysema associated with α_1_-antitrypsin deficiency (AATD, *n* = 23) were used for an exploratory proteomic study aimed at generating fingerprints of these groups that can be used in future pathophysiological and perhaps even clinical research. Liquid chromatography-tandem mass spectrometry (LC-MS/MS) was the platform applied for this hypothesis-free investigation. Analysis of pooled specimens resulted in the production of a “fingerprint” made of 44 proteins for NS/HS; 17 for COPD and 15 for the group of AATD subjects. Several inflammatory cytokines (IL-1α, IL-1β, IL-2; IL-12, α and β subunits, IL-15, interferon α and γ, tumor necrosis factor α); Type I and II cytokeratins; two SP-A isoforms; Calgranulin A and B and α1-antitrypsin were detected and validated through the use of surface enhanced laser-desorption ionization mass spectrometry (SELDI-MS) and/or by Western blot (WB) analysis. These results are the prelude of quantitative studies aimed at identifying which of these proteins hold promise as identifiers of differences that could distinguish healthy subjects from patients.

## 1. Introduction

While blood and urine are the physiological liquids traditionally used for a myriad of tests in humans with different medical conditions, bronchoalveolar lavage fluid (BALF) and induced sputum appear more suitable to monitor the respiratory tract for various compounds [1–13]. Invasive methods of collection may, however, represent an intrinsic limit to the availability of a fluid from patients with poor lung function. In an effort to develop sampling procedures particularly safe and easy to perform on these individuals, several groups are currently exploring the potential of exhaled breath condensate (EBC) as an attractive alternative to BALF and induced sputum [14–19]. Variable amounts of condensate containing volatile and non-volatile compounds may in fact be easily and non-invasively obtained, after cooling water vapor present in exhaled breath, from patients of any age [18]. Since EBC has been demonstrated to derive mainly from the central airways [20], the rationale for its use as a diagnostic tool in clinical analysis is essentially based on the hypothesis that it may contain “biomarkers” *i.e.*, components which are likely to reflect the composition of the airway-lining fluid. However, several methodological questions regarding factors that cause variations in biomarkers’ concentrations represent a potential source of error that may limit the use of this fluid in clinical practice. To minimize such variations, a number of accurate protocols aimed at improving standardization and validation of the method of collection have been published so far [21–24]. Despite these potential drawbacks, EBC has extensively been used for the clinical diagnosis of lung diseases [25], for monitoring of exposure to environmental pollutants or drugs [26], and for studies on lung disorders including asthma, chronic obstructive pulmonary disease (COPD), bronchiectasis, cystic fibrosis (CF) and adult respiratory distress syndrome [25].

With the advent of proteomics, the screening of proteins as potential biomarkers of lung inflammation has achieved marked progress. Detection and identification of proteins in EBC, with the aim of understanding whether they represent an attractive tool for monitoring alterations in the respiratory tract, is currently an area of increasing interest. The utilization of two-dimensional electrophoresis (2-DE), and/or liquid chromatography (LC) and their coupling to mass spectrometry (MS) allowed identification of a series of proteins in EBC [16,18,23]. The application of these techniques in our own laboratory made it possible to show that EBC from patients with α_1_-antitrypsin deficiency-associated pulmonary emphysema contains a higher concentration of protein as compared to controls [27]. Despite this ability of proteomic technology to detect proteins in EBC, reports describing the use of assay platforms for identification of markers that could successfully establish a link between EBC profiles and disease are limited. Obviously, it cannot be expected that monitoring the protein content of EBC samples from individual patients may be initiated without a preliminary extensive investigation to explore (a) which part of the proteome is present in EBC; (b) the variation between sequential samples over a period of time that, although limited, may be relevant to the chronic nature of COPD. Thus, the aim of the current study was to generate a qualitative profile of the proteome in EBC samples obtained from two very distinct clinical phenotypes of COPD and from non-smokers and smokers without COPD to understand whether protein patterns could somehow reflect their lung condition. Liquid chromatography tandem mass spectrometry (LC-MS/MS) allowed producing a tentative “fingerprint” of proteins in EBC of the subjects considered. These results are the prelude of quantitative studies aimed at identifying which of these proteins hold promise to application in monitoring effects of therapeutic interventions.

## 2. Results and Discussion

Demographic data of the 83 subjects considered in this study, classified into different groups as detailed in the experimental section, and protein concentration (μBCA) of EBCs are reported in Table 1.

The age difference between the COPD subjects and the individuals of the other three groups that can be observed in Table 1 could let us hypothesize the generation of results was somehow invalidated by a possible age-bias. Although this bias cannot be excluded “*a priori*”, in our view it is unlikely in this kind of investigation. On the other hand, it should be considered that emphysema associated to smoking and type ZZ α_1_-antitrypsin deficiency occurs at a relative young age compared to subjects without this deficiency, and matching these individuals by age may be a difficult task. Moreover, being the nature of our investigation exploratory, we aimed to study “extremes” of the clinical phenotypes rather than to match them for age.

### 2.1. Sample Pooling

As shown in Table 1, samples were originally collected into four different groups with the aim of performing separate analysis of each of them. However, the LC-MS/MS analysis of individual NS and healthy smokers (HS) samples failed (data not shown). Owing to the paucity of material (<10 μg/mL) mass spectrometry (MS) spectra were of very poor quality and not interpretable for most of them. Sample pooling was considered the only available approach for achieving their enrichment. Thus, samples were grouped on two different items: first, the absence or presence of a reduced forced expiratory volume (FEV1) as the defining character of COPD; second, the presence of an emphysema phenotype *versus* a non-emphysema phenotype. Since cigarette smoking is an important contributor to the pathogenesis of COPD, we considered that smokers with normal FEV1 could be merged with non-smokers. In our opinion a distinction based on normal predicted values (mean ± 2 SD are well established) of FEV1 is justified for a cross-sectional study. Furthermore, our COPD patients had a mean FEV1% of about 45% predicted. This value is well below 60% of the predicted FEV1, a value at which subjects become symptomatic with respect to dyspnea. Thus, whereas pooling samples has the drawback of missing subtle but potentially important differences among subjects, it did allow reliable identification of compounds that could have not been detected in individual samples using current technology.

By contrast, EBCs from α_1_-antitrypsin deficiency (AATD) and COPD, characterized by a higher relative protein concentration (around 20 μg/mL), formed two well-differentiated pools, thus allowing investigating these pathologies separately.

### 2.2. Identification of Proteins with LC-MS/MS

Based upon a series of tryptic matching peptides for each protein analyzed (that ranged between one and 27 and whose primary sequence is included in Table S1 of Supplementary Material), LC-MS/MS allowed the identification of a good number of proteins, including less-abundant ones. A “database” or “fingerprint” was thus produced that contained 44 proteins for the pool of NS/HS, 17 for that of COPD and 15 for that of AATD subjects. It remains a speculation whether the observed differences in the number of proteins between NS/HS and the pools of patients are due to a real effect of disease rather than to different protein concentration and/or to sample handling procedures. If protein concentration is the rationale for a critical analysis of our results, it cannot explain the higher number of proteins in the NS/HS over the AATD pool, with the total protein content of the two pools being comparable (around 370 and 330 μg, respectively). Likewise, it cannot be expected that AATD and COPD pools, which showed a substantial difference in protein concentration (330 *vs.* 210 μg, respectively) could contain a nearly identical amount of proteins. On the other hand, despite having standardized the methods of collection and manipulation, we cannot definitively rule out that the protein composition of a pool may have been somehow altered in response to sample handling procedures. Thus, although many questions are still open, it seems plausible to hypothesize that differences in protein amount between pools may, at least partially, reflect the health state of lung.

All details regarding the identification data of the 44 proteins (including accession number, percent of sequence coverage, number of peptides identified, MOWSE score % and indication of the EBC pool in which each protein was identified) are shown in Table 2.

**Figure 1:**
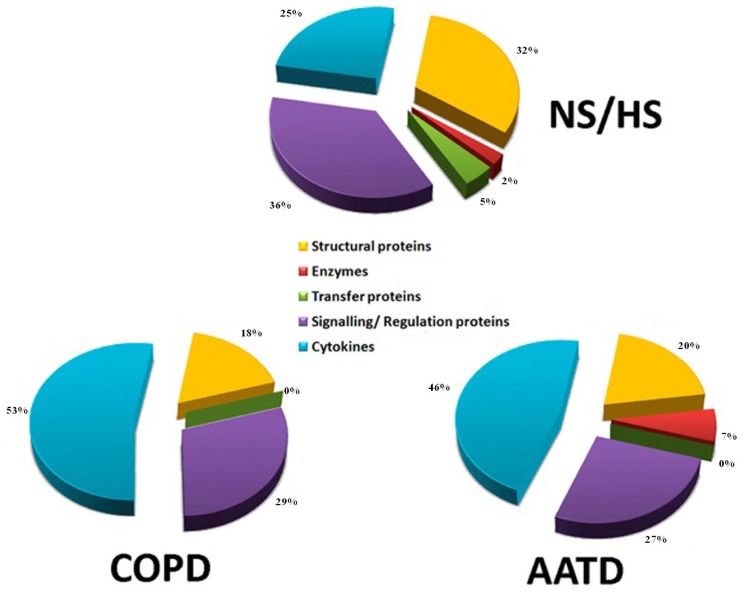
Distribution of identified proteins according to the biological process in which they are involved. Assignments were made on the basis of information provided by GeneOntology (GO) lists downloaded from [28].

Information provided by GeneOntology [28] allowed assigning the distribution of proteins identified according to the biological process in which they were involved (Figure 1). Most proteins in NS/HS were signaling/regulation (34%) and structural (33%), followed by pro-inflammatory (24%), transfer proteins (6%) and enzymes (3%). In COPD (bottom, left) and AATD (bottom, right), cytokines (62%) were predominant over signaling/regulation and structural proteins (15%). Not surprisingly, the burden of cytokines in both groups of patients was much higher than that in controls. Moreover, while AATD contained 8% of enzymes, these were totally absent in COPD subjects. To the best of our knowledge, this is the most complete list of proteins unambiguously and free of hypothesis identified in EBC to date.

### 2.3. Western Blotting and SELDI-MS for Partial Validation of Data

Sodium dodecyl sulfate polyacrylamide gel electrophoresis (SDS-PAGE) followed by Western blot (WB) and surface-enhanced laser desorption ionization mass spectrometry (SELDI-MS) analyses were performed to assess the potential of MS for the tracking of proteins and to validate, at least partially, our findings.

**Figure 2:**
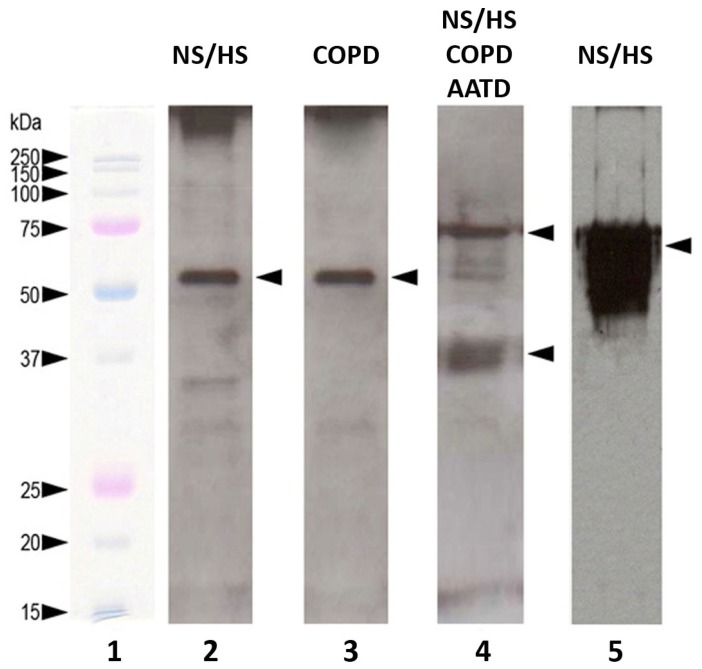
Western blot analysis of the most abundant proteins detected in EBCs analyzed. Lane 1: molecular weight markers; Lanes 2 and 3: α1-antitrypsin (55 kDa band); Lane 4: monomeric (Mr 36 kDa) and dimeric (Mr 72 kDa) surfactant protein A (SP-A); Lane 5: cytokeratin detection in EBC using pancytokeratin antibodies. For more detailed description of procedures, see Experimental Section.

As far as the Western blotting analyses are concerned, we focused our attention on proteins for which monoclonal antibodies were available or whose concentration was sufficient for their detection (*i.e.*, cytokines; cytokeratins; SP-A; α_1_-antitrypsin (AAT)). By incubating polyvinylidene fluoride (PVDF) membranes with antibodies against α_1_-antitrypsin, a single 55 kDa band was observed in EBC samples of NS/HS (Figure 2, Lane 2). The same band was detected in COPD (Lane 3) while no bands appeared in AATD subjects (not shown). Mainly two bands representing monomeric (Mr 36 kDa) and dimeric surfactant protein A (SP-A) (Mr 72 kDa) were noted in EBC of NS/HS, as well as of COPD and AATD (Lane 4). Western blots were also developed for cytokeratin detection in EBC using pancytokeratin antibodies. Only the NS/HS group was positive in this assay showing the large band that can be observed in Lane 5 of Figure 2.

**Figure 3:**
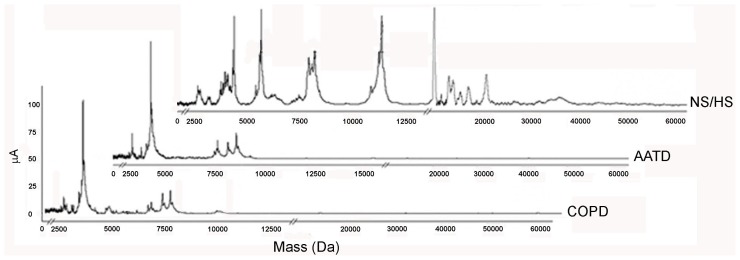
Top to bottom: surface-enhanced laser desorption ionization mass spectrometry (SELDI) profiles of EBC from NS/HS, AATD and COPD subjects. These patterns are representative, for each group, of all those produced on the CM 10 weak cation exchanger. For additional details refer to the experimental section.

As shown in Figure 3, the SELDI profiles generated by spotting EBC samples on the CM 10 weak cation exchange array (according to the rationale described in details in Paragraph 3.4), contained a number of *m*/*z* signatures much higher for NS/HS subjects than for AATD and COPD patients (top to bottom, respectively). Provided that each signature could correspond to a protein, these results were somehow in agreement with the LC-MS findings previously observed in Table 2.

From among the *m*/*z* SELDI signatures observed, 27 (listed in Table S2 of Supplementary Material) revealed an excellent matching with the theoretical *M*_r_ values of proteins identified by LC-MS/MS.

**Figure 4:**
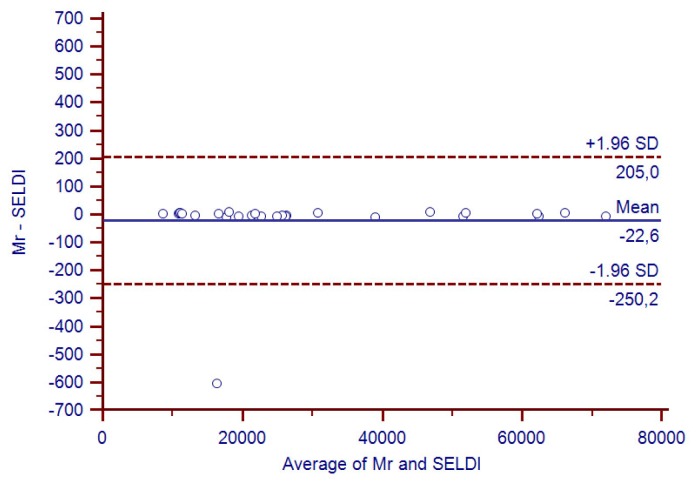
Open circles show the Bland-Altman agreement analysis between the 27 SELDI *m/z* signatures, which matched the proteins identified by Liquid chromatography-mass spectrometry (LC-MS) and their theoretical *M*_r_ value.

The correlation between these values, plotted according to the Bland-Altman’s [29] model, is shown in Figure 4. While not being “*per se*” a reliable criterion for assessing the identity of proteins (only the mass of a protein is provided by SELDI), such a good concordance was highly unlikely to be due to chance and strengthened our opinion that the platform applied was indeed a reliable tool for identifying proteins in EBC.

### 2.4. Identification of Proteins Contained in EBCs

The most abundant proteins found in LC-MS were Type I and II cytokeratins: CK-1; CK-5; CK-9; CK-14 and CK-26. The corresponding SELDI signatures (in growing order of their *m*/*z*) were: 51,568 (CK-14); 51,906 (CK-26); 62,061 (CK-9); 62,384 (CK-5) and 66,032 (CK-1) Da. A number of these CK proteins had previously been identified in EBC by other authors and also by us [16,18,27,30]. Recently it has been speculated that these proteins can be contaminants [31] or may have exogenous origins and are probably retained by the respiratory system [24]. Although every possible precaution was taken to avoid contamination, obviously the risk that exogenous keratins may have been somehow introduced into the samples (in the course of the analytical procedures) cannot be excluded. With respect to our experimental setting, we note that (i) while having performed independent replicates, cytokeratins (CKs) were not spread out across our entire set of samples; (ii) tryptic peptides matched well-identified keratins with an excellent score (91% to 99%); (iii) three keratins out of five were the same as those previously identified [27]. In addition, Cytokeratins 5 and 6, expressed by the bronchial epithelium, have been suggested to serve as possible indicators of lung damage inflicted by emphysema in COPD [32]. The finding of CKs mainly in EBC of HS/NS seems to be contradictory with this hypothesis, unless they are actually a contamination introduced during the initial HS/NS enrichment step. If not, one explanation for their finding in controls only could be that EBC of COPD patients contains a higher protein load (in terms of concentration) due to the presence of many inflammatory proteins which mask the relatively low content of cytokeratins. In view of the importance of these compounds, further studies will be carried out to obtain more precise information on their function in the pathogenesis of lung diseases and their value as diagnostic or prognostic markers.

Several inflammatory cytokines (IL-1α, IL-1β, IL-2; IL-12, α and β subunits, IL-15, interferon α and γ, tumor necrosis factor α) and complement C3 were detected. Most of them were confirmed also by SELDI signatures (*m*/*z* at 21,722 and 19,354 Da for interferon α and γ, respectively; 25,647 Da for tumor necrosis factor; 30,743 Da for IL-1β; 17,636 Da for IL-2; 24,879 Da for IL-12, α subunit; 18,078 Da for IL-15). They were predominant over other proteins in EBC of patients but not in that of NS/HS. Many of these proteins have previously also been detected by other authors [33–38]. Their finding by MS methods is remarkable since the identification of cytokines in EBC had not been definitively demonstrated yet, owing to their concentration that is often close to the lower limit of detection of immunochemical methods currently applied for their measurement [39]. With the exception of TNF and IL-15, for which the MOWSE score was low, other cytokines have been identified unambiguously. While interferon α1 and IL-12 were identified only in COPD, IL-1α was detected in the AATD group only. This result is of particular interest because it could allow the discrimination between the two groups of patients. Also lysozyme C (*m*/*z* signature at 16,533 Da), an enzyme associated with the monocyte-macrophage system that enhances the activity of immunoagents, was detected in the AATD with emphysema patients only. The isoforms of surfactant protein A (SP-A1 and SP-A2) were identified with a 98% score in the three pools analyzed. Despite the high sequence similarity between these two isoforms, the finding of a tryptic peptide (HQILQTR, see Table S1) specific for SP-A1 allowed to discriminate between the two and emphasized, if necessary, the great potential of MS methods. The presence of two SELDI signals with appropriate *m*/*z* (26,245 and 26,187 Da for SP-A1 and SP-A2, respectively) and the observation on the Western blotting (Figure 2, lane 3) of two bands representing monomeric and dimeric SP-A unambiguously confirmed the LC-MS data. Simpson *et al.*[12] first assessed SP-A in EBC of non-smoking adults with asthma. The perfect correspondence of SELDI signals (*m*/*z* at 10,833 and 13,245 Da) with the molecular weight of Calgranulin A and B (10,835 and 13,242 Da, respectively) was decisive to confirm their presence in EBC of all subjects. The identification of these two calcium-binding proteins confirmed previous data from other authors [18]. Among the signaling/regulation proteins, the identification with a good (97%) MOWSE score of α1-antitrypsin in NS/HS and COPD groups was of great interest. The best candidate SELDI ion signature associated with this protein was that with *m*/*z* at 46,728 Da. Unambiguous confirmation of its presence was provided by immunochemical analysis (Figure 2, Lanes 2 and 3). As expected, this signal was not found in EBC of AATD patients. To our knowledge detection of AAT in EBC has been reported only in another very recent study [40].

The SELDI *m*/*z* signatures of Histone H 1.5 (22,586 Da); Erythropoietin (21,311 Da); Protein α-1-Microglobulin/Bikunin Precursor (39,009 Da); Ubiquitin (8,562 Da); Cystatin (11,001 Da); Kininogen 1 (71,963 Da); Monocyte Chemotactic Protein-1 (11,020 Da); Growth-regulated oncogene alpha protein (11,298 Da); Hemoglobin subunit beta (16,601 Da) confirmed the LC-MS findings. Given its function, Monocyte Chemotactic Protein-1 (MCP-1) seems particularly interesting among the proteins indicated above. This protein is produced predominantly by macrophages and endothelial cells and is a potent chemotactic factor for monocytes MCP-1. It recruits monocytes, memory T cells and dendritic cells to sites of tissue injury and infection and, for this reason, it could be of great interest to monitor its expression in the course of disease progression.

The identification in EBC of the NS/HS pool of Hemoglobin, subunit-β, is also noteworthy. Interestingly, this protein has previously been observed in serum by Gomes-Alves *et al.*[41]. Comparing the surface enhanced laser desorption/ionization time-of-flight (SELDI-TOF) profiles from serum of cystic fibrosis, asthma and COPD patients, they found β-Hb to be differentially expressed in CF patients compared to asthma and control subjects. Given that all samples that presented hemolysis had previously been discarded, they hypothesized, also on the basis of other studies [42], that β-Hb was the result of a hemolytic process due to oxidative stress on erythrocytes. Indeed oxidative stress, which has been shown to be implicated in the pathogenesis of pulmonary emphysema [43,44], could also be responsible for the presence of β-Hb in EBC. In our case, a rich source of oxidants is, obviously, cigarette smoke. Oxygen radicals, with their capability of modifying amino acid side chains, form protein aggregates, cleave peptide bonds and make proteins more susceptible to proteolytic degradation and may cause hemolysis of erythrocyte membrane and release β-Hb. However, it remains unclear why this protein was found in NS/HS only.

### 2.5. Study Limitations

This work has some limitations that should be overcome with appropriate future investigations. First of all, individual samples could not be analyzed but they were pooled for each group and, in one case, namely NS and HS, two groups were pooled together. While being a possible source of bias, this procedure was necessary to focus on the question of whether or not EBCs from different classes of individuals may contain specific proteins. Contrasting results that have appeared in the literature in the recent past in fact leave this question still unanswered. Efforts will be done in future work to collect sufficient material to analyze these two groups separately. The second limitation of our work is the lack of an extensive validation of results. Although SDS-PAGE/WB and SELDI-MS have provided a partial confirmation of proteins detected by LC-MS/MS, we are aware that these data do not represent robust evidence. A third limitation is the lack of quantitative results, although this is strictly related to the exploratory nature of the work. With the aim of producing a preliminary fingerprint of proteins that could reflect the health state of the lung, we focused on the qualitative aspect of the research only. Provided that our data are a reliable fingerprint, it will be of great interest to understand which of these proteins/groups of proteins is over/under expressed in patients compared to controls.

## 3. Experimental Section

### 3.1. Study Design and Study Population

The study had a cross-sectional design and all measurements were performed within two days. A history and physical exam was taken from all subjects. Individuals who reported no respiratory symptoms, who had a normal physical exam and who had normal spirometry were considered healthy. Spirometry was performed according to European Respiratory Society (ERS) guidelines, and the presence or absence of emphysema in subjects with COPD was measured by computed tomography of the chest as previously described. A visual scoring as described previously [45] was used to confirm the absence of emphysema in the COPD patients. The presence of emphysema in subjects with genotype ZZ of alpha-1-antitrypsin deficiency was confirmed by the same method. EBCs were collected from 83 subjects classified into four groups. Group 1 (“NS”; *n* = 25; 12 men and 13 women; mean age 33 ± 4.5 years) were never-smoking volunteers (controls) with no known respiratory disease and free from symptoms of respiratory tract infection or medication use at least four weeks prior to the study. They had normal spirometry according to ERS guidelines [45]. Group 2 (“HS”; *n* = 20; 10 men and 10 women; mean age 35 ± 4.0 years) were asymptomatic smokers with neither airflow obstruction as assessed by spirometry nor mucus hypersecretion. Sex and, when possible, age of subjects belonging to these two groups were matched to patients enrolled in the two other groups. Group 3 (“AATD”; *n* = 23; 11 men and 12 women; mean age 40 ± 2.5 years) were subjects who suffered from pulmonary emphysema associated with α_1_-antitrypsin deficiency. The presence of airflow obstruction (FVC/FEV_1_ < 70% and FEV_1_ 46 ± 4% predicted, Table 1) but no emphysema at high resolution computed tomography, as assessed by a radiologist, defined Group 4 (“COPD”; *n* = 15; 7 men and eight women; mean age 65 ± 8 years; FVC/FEV_1_ < 70% and FEV_1_ 47 ± 9% predicted). All patients were neither on aspirin nor on non-steroidal anti-inflammatory agents or systemic steroids. They also had neither intercurrent infection nor exacerbation in four weeks prior to sampling EBC. Each participant had baseline spirometry to measure FEV_1_, forced expiratory capacity (FVC) and FEV_1_/FVC. Subjects were enrolled by the Pulmonary Department of the Leiden (The Netherlands) University Medical Centre. Subjects recruited gave their informed consent, and the investigation was approved by the local ethical board.

### 3.2. EBC Collection and LC-MS/MS Analysis

Exhaled breath condensate was collected by using the RTube kit (Respiratory Research Inc., Austin, TX, USA) according to ATS/ERS guidelines [21]. The volume of condensate collected was typically 1.2 ± 0.3 mL for controls and ranged between 650 and 850 μL for both AATD and COPD patients. Salivary contamination was checked by measuring the α-amylase activity in all samples with the α-amylase ESP 1491300 kit (Boehringer-Ingelheim, Mannheim, Germany) whose detection limit was 0.003 U/mL. No amylase activity was detected in samples considered for this investigation.

Samples were dried under vacuum in a Speed Vacuum concentrator (Thermo Fisher Scientific Inc., Southend-on-sea, UK), reconstituted in 100 μL of 50 mM ammonium bicarbonate pH 8.5 containing 100 mM DTT and treated with sequencing grade trypsin at a concentration of 10 mg/mL in a 50:1 ratio. Tryptic digestion was performed overnight at 37 °C and peptides were separated by reversed phase high performance-liquid chromatography (RP-HPLC) on a Phenomenex (Torrance, CA, USA) Jupiter C18 column with 150 × 2 mm, 4 μm (90 Å particle size), flow-rate 0.2 mL/min and on a 150 × 0.3 mm column, 4 μm (90 Å particle size), flow-rate 500 nL/min, using a linear gradient (2% to 70% solvent B in 90 min) in which solvent A consisted of 0.1% aqueous formic acid (FA) and solvent B of acetonitrile (ACN) containing 0.1% FA. Identification of biomarkers in EBC samples was achieved by means of (i) a liquid chromatography-tandem mass spectrometer (LC-MS/MS, Thermo Finnigan, San Josè, CA, USA) system coupled to a Linear Trap Quadrupole (LTQ) mass spectrometer with electrospray ionization (ESI) ion source controlled by Xcalibur software 1.4, and (ii) an UltiMate 3000 system (Dionex) coupled to a LTQ Orbitrap mass spectrometer with nano-ESI ion source controlled by Xcalibur software 2.0.7. (Thermo Fisher Scientific Inc.: Southend-on-sea, UK).

All mass spectra were generated in positive ion mode under constant instrumental conditions: source 4.0 kV, capillary voltage 46 v, sheath gas flow 40 (arbitrary units), auxiliary gas flow 10 (arbitrary units), sweep gas flow one (arbitrary units), capillary temperature 250 °C, tube lens voltage −105 V. MS/MS spectra, obtained by CID studies in the linear ion trap, were performed with an isolation width of 3 Th *m/z*, the activation amplitude was 35% of ejection RF amplitude that corresponds to 1.58 V.

Data acquisition and processing were performed using Peaks studio 4.5 (Bioinformatics Solutions Inc.: Waterloo, ON, Canada) and Bioworks version 3.1 (Thermo Fisher Scientific Inc.: Southend-on-sea, UK) softwares. The mass lists were searched against the SwissProt protein database using MASCOT [46] search engine under continued mode (MS plus MS/MS) with the following parameters: trypsin and chymotrypsin specificities, five missed cleavages, cysteine carbamido-methylation as variable modifications, peptide tolerance at 0.2 Da and MS/MS tolerance at 0.25 Da. Peptide charge 1, 2, 3 + and monoisotopic mass values were considered. MASCOT scores greater than 65 were considered significant (*p* < 0.05).

### 3.3. One-D PAGE and Western Blotting

A pellet from EBC samples was reconstituted in 30 μL of 1 M Tris–HCl pH 6.8 containing 5% 2-mercaptoethanol, 2% sodium dodecylsulphate (SDS), 0.02% bromophenol blue (BPB) and 10% glycerol. Samples were incubated at 100 °C for 5 min and then loaded on gel slabs.

Electrophoresis was performed in 5% stacking gel and 15% running gel by applying a voltage of 150 V for 1 h. Gels were stained with silver nitrate. Blotting on Millipore PVDF membranes (Billerica, MA, USA) was performed at 250 mA for 2 h in transfer buffer (25 mM Tris, 192 mM glycine, pH = 10.3, containing 20% methanol). Membranes were blocked in 5% of non-fat dry milk in PBS-buffer containing 0.1% Tween-20 o/n at 4 °C on rollerbank and were incubated for 2 h in the presence of primary antibodies diluted 1:1000/2000 in blocking buffer (goat anti-human SP-A and anti-human α_1_-AT, mouse anti-human cytokeratins). Subsequently they were washed in PBS-buffer containing 0.05% Tween-20 and reacted for 1 h with the rabbit anti-goat Ig secondary antibody conjugated with HRP diluted 1:1000. After washing in the same buffer as indicated above, membranes were incubated for 2 min in 4 mL of ECL substrate (Pierce) and finally proteins visualized on films.

### 3.4. SELDI-TOF Analysis

With the aim of capturing the largest possible number of EBC proteins, four chip array surfaces characterized by distinct adsorption properties (weak cation exchanger CM 10; anion exchanger Q 10; normal phase NP 20; reverse phase H 50), were screened. The results provided by the hydrophobic surface H 50 and the anion exchanger Q 10 were discouraging. No peaks at all were observed by spotting samples on the former and very few *m*/*z* signatures (characterized by a S/N ratio <4) appeared when using the latter. A higher amount of material loaded on the chip did not result in any improvement of signals thus suggesting that very few, if any, proteins were retained by these surfaces. Being not informative, these two arrays were excluded from further investigations, also in the light of the results obtained by applying the same samples on the hydrophilic-interaction surface NP 20 and on the weak cation exchanger CM 10. Given the close chemical characteristics of the surfaces involved, these two arrays generated profiles very similar in terms of number of signals observed *per* group and of their size distribution. However, while the number of peaks with S/N ratio > 4 was similar in both profiles, the intensity of signals of the NP 20 surface appeared much lower compared to that of the CM 10. Thus, based on the conviction that the analysis of patterns containing high-intensity signals could maximize the chance to detect differential profiles among groups, the CM 10 profiles were chosen as the “model-profiles” of EBCs investigated. All 83 specimens were processed in triplicate and analyses resulted in protein profiles characterized by masses ranging from 2 to 67 kDa for all subjects. In brief, each pool was ten-fold concentrated under vacuum in a Speed Vacuum concentrator (Thermo Fisher) and one μL was withdrawn, applied onto a chip (CM 10), dried and washed (twice) with water according to the Ciphergen protocol. Dried spots were then coated with two 0.5 μL additions of matrix solution (CHCA; α-cyano-hydroxycinnamic acid, Ciphergen) in 0.5% *v*/*v* trifluoro acetic acid (TFA), 50% acetonitrile (ACN). The arrays were read on a Protein Biosystem II (Ciphergen) instrument. Spectra were collected automatically using the following instrumental parameters: range 1000–70,000 Da; focus mass 10 kDa; laser energy 3000 nJ; detector sensitivity 3655 shots kept. External calibration of the instrument was performed by using the All-in-One peptide and the All-in-One protein molecular mass standards II (Ciphergen) on the NP 20 array. All spectra were calibrated by using the equation: (*m*/*z*)/U = 3.29 E8 (t + 3.34 E8). Only signals with a S/N > 4 were taken into consideration. To verify reproducibility of data, samples were processed in triplicate.

### 3.5. Reproducibility of the Study

To check the reproducibility of the study, LC-MS and SELDI analyses were performed in triplicate. Identification of protein in LC-MS or *m*/*z* signature in SELDI was considered reliable only if found in two over the three attempts performed.

The agreement between LC-MS and SELDI data was assessed using the Bland-Altman model [29].

## 4. Conclusions

Through the combination of current MS techniques, this exploratory study resulted in the generation of a panel of EBC proteins, which are supposed to originate directly from the airways and the lung. It is still unclear how representative this proteome is. In other words the question of whether only the most abundant proteins have been systematically identified and whether special challenges should be posed for increasing detection capabilities of less abundant ones, still has to be answered. With respect to the proteins identified, while not all of them are expected to be unique to pulmonary disorders, it is essential to understand which specific combination could possibly represent COPD and/or emphysema. The set of proteins that would monitor the disorder could thus become the classifier for discriminating controls from patients. It is noteworthy that all proteins identified in the COPD and AATD groups were present also in NS/HS group. Thus, any investigation that addresses the quantitative assessment of these proteins will allow monitoring of protein expression levels and hopefully provide decisive progress for the identification of a biomarker of the disease.

## Supplementary Information



## Figures and Tables

**Table 1:** Demographic Table showing the characteristics of subjects investigated.

Subjects	Smoking status	Age (Years)	FEV_1_* (L)	FEV_1_ (%)	FVC ** (L)	FEV_1_/FVC (%)	Protein concentration in EBC (μg/mL)
NS (*n* = 25; F/M = 13/12)	Never-smokers	33 ± 4.5	3.05 ± 0.67	91.52 ± 2.92	3.32 ± 0.79	75 ± 3.37	6.8 ± 0.7
HS (*n* = 20; F/M = 10/10)	Smokers (8 p/y)	35 ± 4.0	2.94 ± 0.36	89.45 ± 4.28	3.16 ± 0.51	72 ± 2.95	7.1 ± 0.9
COPD (*n* = 15; F/M = 8/7)	Ex-smokers	65 ± 8.0	1.56 ± 0.39	47.44 ± 9.37	1.84 ± 0.92	45 ± 9.42	19.3 ± 0.5
AATD (*n* = 23; F/M = 12/11)	Ex-smokers	40 ± 2.5	1.34 ± 0.51	45.92 ± 3.71	1.57 ± 0.58	43 ± 5.67	21.7 ± 0.4

* Forced expiratory volume in the first second;

** Forced vital capacity.

**Table 2:** List of proteins identified in exhaled breath condensates (EBCs) of subjects analyzed, accession number, Mr, percent of sequence coverage, number of peptides identified, MOWSE score % and indication of the EBC pool in which each protein was identified.

#	Description	Accession #	Mr (Da)	ESI Ion-trap/Nano ESI orbitrap			Presence in		
	
				Coverage%	Query	MOWSE score%	NS/HS	COPD	AATD
1	Type II, CK 1	P04264	66039	16/25	12/20	99/99	√	—	—
2	Type II, CK 2	P35908	65433	5/24	4/15	91/99	√	—	—
3	Type II, CK 5	P13647	62378	34/23	119/13	99/99	√	—	—
4	Type II, CK 6B	P04259	60067	35/55	99/96	99/99	√	—	—
5	Type I, CK 9	P35527	62064	42/11	101/10	99/99	√	—	—
6	Type I, CK 10	P13645	58827	44/19	69/15	99/99	√	—	—
7	Type I, CK 14	P02533	51562	36/12	58/9	99/99	√	—	—
8	Type I, CK 26	Q7Z3Y9	51911	37/12	68/5	99/82	√	—	—
9	ACTC1	P68032	42019	24/5	48/2	67/90	√	—	—
10	PS-A1	Q8IWL2	26242	30/3	12/2	98/93	√	√	√
11	PS-A2	Q8IWL1	26182	39/2	14/1	98/90	√	√	√
12	HSPG	P98160	468803	12/4	60/22	98/99	√	—	—
13	LAMB4	B4DX23	76746	39/10	161/9	99/90	√	—	—
14	Histone H1.5	P16401	22580	85/32	197/8	99/99	√	√	√
15	Alpha-1-AT	P01009	46737	41/5	24/2	97/82	√	√	—
16	Calgranulin A	P05109	10835	68/16	18/2	61/76	√	√	√
17	Calgranulin B	P06702	13242	16/15	8/2	51/72	√	√	√
18	Erythropoietin	P01588	21307	20/5	14/2	98/83	√	—	—
19	Protein AMBP	P02760	39000	22/15	20/8	59/85	√	—	—
20	Ubiquitin	P62988	8565	43/16	8/3	92/81	√	—	—
21	Cystatin	P01040	11006	20/20	7/3	36/63	√	√	√
22	CSAct	P07602	58113	24/11	10/5	99/88	√	—	—
23	LAMP2	P13473	44961	18/16	8/10	24/99	√	—	—
24	Kininogen 1	P01042	71957	31/17	41/12	99/91	√	—	—
25	Complement C3	P01024	187147	27/6	76/11	99/99	√	—	—
26	Nucleolar protein 4	O94818	58419	23/4	117/3	99/95	√	—	—
27	VSIG8	Q5VU13	43891	18/6	46/1	96/90	√	√	√
28	THRAP3	Q9Y2W1	108666	57/36	421/44	99/99	√	—	—
29	CHD1	O14646	196688	41/20	432/45	99/99	√	—	—
30	ZC3H4	Q9UPT8	140257	23/11	240/21	99/99	√	—	—
31	MCP-1	P13500	11025	38/7	12/3	95/85	√	√	√
32	IFN α-1/13	P01562	21725	17/9	13/6	96/86	√	√	—
33	IFN γ	P01579	19348	36/22	27/18	99/93	√	√	√
34	TNF	P01375	25644	20/11	7/5	28/64	√	√	√
35	GRO-α	P09341	11301	73/71	19/9	85/99	√	√	√
36	IL-1 α	P01583	30607	15/—	8/—	90/—	√	—	√
37	IL-1 β	P01584	30748	28/7	23/3	94/60	√	√	—
38	IL-2	P60568	17628	32/9	10/5	99/79	√	√	√
39	IL-12 subunit α	P29459	24874	30/8	10/2	92/80	√	√	—
40	IL-12 subunit β	P29460	37169	30/15	26/13	98/83	√	—	—
41	IL-15	P40933	18086	7/—	2/—	52/—	√	√	√
42	Hb subunit β	P68871	15998	2/16	1/2	90/88	√	—	—
43	Serum Albumin	P02768	69367	34/25	44/13	98/94	√	—	—
44	Lysozyme C	P61626	16537	42/13	31/6	99/94	√	—	√
