# Supplementary material for: Profiling the Proteome of Exhaled Breath Condensate in Healthy Smokers and COPD Patients by LC-MS/MS
Source: Int J Mol Sci. 2012 Oct 29;13(11):13894–910. doi: 10.3390/ijms131113894 (PMC3509556; doi:10.3390/ijms131113894)
Supplement: Supplementary file 1 [file ijms-13-13894-s001.pdf]

Supplementary Information

Table S1. Primary sequence of peptides found for each protein identified in EBCs. Numbers between brackets indicate the number of times that peptide was identified. The start-end sequence of each peptide is also indicated.

#	Description	Accession n°	Sequence	m/z	z	Start	End
1	Keratin, type II cytoskeletal 1	P04264	VDLQAK	337.37225	2	299	304
			NKYEDEINKR	437.21942	3	268	277
			SKAEAESLYQSK (x2)	671.00305	2	365	376
			GGGGGGYGSGGSSYGSGGGSYGSGGGGGGGR	1192.5554	2	519	549
			AQYEDIAQK	533.57104	2	356	364
			TLLEGEESR (x2)	517.3922	2	484	492
			SISISVAR (x2)	417.00964	2	75	82
			WELLQQVDTSTR (x2)	738.4602	2	212	223
			FLEQQNQVLQTK	738.6218	2	200	211
			YEELQITAGR (x2)	590.53296	2	377	386
			TNAENEFVTIK	633.4143	2	278	288
			NMQDMVEDYR	650.99744	2	258	267
			SLNNQFASFIDK (x3)	692.46375	2	186	197
			SLDLDSIIAEVK (x2)	651.9164	2	344	355
			QFSSR	624.3263	1	4	8
2	Keratin, type II cytoskeletal 2	P35908	FLEQQNQVLQTK (x2)	738.4602	2	198	209
			VDLLNQEIEFLK	731.13257	2	303	314
3	Keratin, type II cytoskeletal 5	P13647	NLDLDSIIAEVK	664.75321	2	332	343
			QLDSIVGER (x3)	508.0647	2	229	237
			SLYNLGGSK (x2)	938.03946	1	64	72
4	Keratin, type II cytoskeletal 6B	P04259	GFSANSAR	808.8635	1	17	24
			ATGGGLSSVGGGSSTIK (x4)	717.5847	2	534	550
			SEIDHVK (x2)	826.0647	1	393	399
			QNLEPLFEQYINNLR	945.4821	2	208	222
5	Keratin, type I cytoskeletal 9	P35527	EVTQLR	373.36237	2	369	374
			GGSGGSYGGGGSGGGYGGGSGSR	896.6017	2	491	513
			FSSSSGYGGGSSR (x2)	618.3602	2	47	59
			STMQELNSR (x2)	533.386	2	155	163
			QEYEQLIAK	561.4629	2	328	336
			QGVDADINGLR	579.39014	2	251	261
			TLLDIDNTR	531.0093	2	225	233
			MTLDDFR	449.39258	2	234	240
			LASYLDKVQALEEANNDLENK	793.3523	3	164	184
			QFSSSYLSR (x12)	537.4099	2	5	13

Table S1. *Cont.*

#	Description	Accession n°	Sequence	m/z	z	Start	End
6	Keratin, type I cytoskeletal 10	P13645	SQYEQLAEQNRK (x2)	747.6277	2	323	334
			IKEWYEK	498.49396	2	178	184
			VTMQNLNDR (x3)	546.0193	2	148	156
			LAADDFR	404.37274	2	229	235
			LASYLDKVR (x2)	532.91284	2	157	165
			ALEESNYELEGK (x2)	691.5386	2	166	177
			IRLENEIQTYR	718.18567	2	440	450
			QSVEADINGLR	601.51807	2	246	256
			QSLEASLAETEGR (x2)	696.01025	2	387	399
			VLDELTLTK (x2)	516.3241	2	258	266
			DAEAWFNEK (x2)	555.43396	2	335	343
			NVSTGDVNVEMNAAPGVDLTQLLNNMR (x3)	1436.707	2	296	322
			DYSK	256.41565	2	195	198
7	Keratin, type I cytoskeletal 14	P02533	GSCGIGGGIGGGSSR (x2)	610.4852	2	16	30
			ILNEMR (x3)	774.3254	1	283	288
			LLEGEDAHLSSSQFSSGSQSSR	769.7523	3	418	439
8	Keratin, type I cytoskeletal 26	Q7Z3Y9	VTMQNLNDR (x3)	546.0193	2	148	156
9	ACTC1	P68032	VAPEEHPTLLTEAPLNPK	978.3625	2	98	115
			EITALAPSTMK (x4)	580.5421	2	318	328
			ILTER (x2)	630.7589	1	194	198
10	Pulmonary surfactant-associated protein A1	Q8IWL2	NPEENEAIASFVK	723.7825	2	167	179
			GEPGER (x2)	643.6542	1	89	94
			HQILQTR	447.5145	2	116	122
			EQCVEMYTDGQWNDR (x2)	936.9931	2	222	236
			YSDGTPVNYTNWYR (x2)	867.9154	2	200	213
11	Pulmonary surfactant-associated protein A2	Q8IWL1	NPEENEAIASFVK (x2)	723.7825	2	167	179
			GEAGER	617.6163	1	89	94
			IAVPR (x3)	554.6907	1	162	166
			EQCVEMYTDGQWNDR	936.9931	2	222	236
12	HSPG	P98160	GGNLPAR (x3)	684.13416	1	2864	2870
			SQSVRPGADVTFICTAKSK	997.95935	2	1780	1798
			HQIVGSRLR (x11)	533.4254	2	2578	2586
			YNVR (x9)	275.70428	2	616	619
			LLSR (x18)	488.46674	1	644	647
			WHRVSAERLNKDGSLRVNGGRPVLR (x2)	1436.8923	2	4013	4037
			GGSLPSHHQTRGSR (x7)	738.6218	2	2764	2777
13	LAMB4	B4DX23	LAGDTEAK	803.8663	1	672	679
			VANGVLDIHLPIPSQNLTDELVK (x3)	828.3327	3	471	493
			AGTQKPICDPDTGMCR (x5)	846.9861	2	102	117
			EGVSGQR (x2)	731.5544	1	120	126
14	Histone H1.5	P16401	NGLSLAALK (x2)	443.4723	2	58	66
			AAAKPK (x6)	584.1452	1	179	184
			MSETAPAETATPAPVEK (x3)	864.4721	2	1	17

Table S1. *Cont.*

#	Description	Accession n°	Sequence	m/z	z	Start	End
15	Alpha-1-antitrypsin	P01009	WERPFEVK (x3)	546.0193	2	218	225
			LSITGTYDLK (x2)	555.43396	2	315	324
			ELDR (x16)	532.2914	1	199	202
			AVHK (x2)	453.3944	1	356	359
			DTEEEDFHVDQVTTVKVPMMKR	1317.178	2	226	247
16	Calgranulin A	P05109	GNFHAVYRDDLK (x2)	718.18567	2	24	35
			KSHEESHKE (x3)	555.43396	2	85	93
			YSLIK	624.3263	1	19	23
17	Calgranulin B	P06702	ELVR (x3)	515.4436	1	39	42
			DLQNFLKK	503.1975	2	44	51
			ELVRKDLQNFLK	752.3341	2	39	50
18	Erythropoietin	P01588	LYTGEACRTGDR (x3)	671.00305	2	182	193
			LKLYTGEACR (x2)	577.55273	2	180	189
			YLLEAK	736.2893	1	42	47
			VLER	515.4547	1	38	41
			VYSNFLR (x10)	449.32288	2	171	177
19	Protein AMBP	P02760	GECVPGEQEPEPILIPRVRR	758.5582	3	186	205
			ETLLQDFR (x3)	511.52173	2	159	166
			LYGRAPQLR (x14)	537.5405	2	150	158
			FYSEKECR (x2)	1061.177	1	327	334
			KFSRHHGPTITAKLYGR (x2)	984.57837	2	137	153
			MTVSTLVLGEGATEAEISMTSTR	1192.5554	2	63	85
20	Ubiquitin	P62988	LRGG (x2)	401.29382	1	73	76
			LIFAGK	647.4166	1	43	48
			MQIFVKTLTGK	633.4143	2	1	11
			AKIQDK (x3)	702.0874	1	28	33
			TLSDYNIQK	1082.3425	1	55	63
21	Cystatin	P01040	AGDNK (x10)	252.62006	2	59	63
			YMHLKVFK (x9)	533.386	2	64	71
			AGDNKYMHLKVFK (x3)	775.86584	2	59	71
			DDELTGF (x3)	796.40625	1	92	98
22	CSAct	P07602	LIDNNKTEK (x13)	537.5405	2	328	336
			GSAVWCQNVK (x3)	546.0193	2	28	37
			MCSK (x11)	234.8487	2	345	348
			SKPQPK (x3)	684.3153	1	187	192
			LGPGMADICK	503.1975	2	233	242
23	LAMP2	P13473	NENR (x17)	532.39343	1	290	293
			WQMNFTVR (x4)	1082.3425	1	46	53
			VASVININPNTTHSTGSCRSHTALLR	1375.5791	2	248	273
24	Kininogen 1	P01042	NKGKKNGK (x5)	437.3499	2	508	515
			RHDWGHEKQRK (x6)	738.4602	2	428	438
			RPPGFSPFRSSR (x2)	696.01025	2	381	392
			IGEIK (x5)	559.3855	1	393	397
			VQVVAGKK (x2)	414.87122	2	309	316
25	Complement C3	P01024	QNQELK	379.90448	2	856	861
			QPSSAFAAFVKR	437.21942	3	1061	1072
			FLTTAK	679.626	1	1210	1215
			EGVQK (x6)	280.76257	2	955	959
			QHAR (x8)	256.34106	2	737	740
			QLANGVDR (x2)	437.09894	2	1420	1427
			VTLEER	373.36237	2	1527	1532

Table S1. *Cont.*

#	Description	Accession n°	Sequence	m/z	z	Start	End
26	Nucleolar protein 4	O94818	TGLEQDEQPLNLSDSPLSAQLTSEYR (x2)	964.2254	3	295	320
			QQDESAPADK	544.2687	2	513	522
			QFQDWCLR (x4)	547.5421	2	10	17
			EAVTR	574.5412	1	149	153
27	VSIG8	Q5VU13	SAEPADCAEGPVQCK (x5)	752.6621	2	395	409
			INGDGQEVLYLAEGDNVR	981.4475	2	24	41
			TTMATR (x3)	679.7722	1	131	136
28	THRAP3	Q9Y2W1	GFYPWGQYNR	643.4562	2	92	101
			SPSELFAQHIVTIVHHVK (x4)	1021.3257	2	622	639
29	CHD1	O14646	EYTNPEQIK	560.5214	2	1464	1472
			EDDSNLLIGIYEYGYGSWEMIK (x2)	865.2269	3	1256	1277
			LLITGTPLQNSLK (x7)	698.4587	2	639	651
30	ZC3H4	Q9UPT8	EGEQDAASLK (x4)	523.4687	2	1279	1288
			HHSDSDEEK	541.3278	2	90	98
			ELNQYR (x2)	821.9112	1	223	228
			LYHTTGNCINGDDCMFSHDPLTEETR (x6)	990.0665	3	450	475
31	MCP-1	P13500	ITSSKCPKEAVIFK (x3)	775.86584	2	54	67
			CPKEAVIFK (x2)	517.3922	2	59	67
			KISVQRLASYRR (x5)	738.4602	2	42	53
			TIVAK (x8)	532.2914	1	68	72
32	Interferon alpha-1/13	P01562	LRRKE (x5)	701.88586	1	185	189
			YFRR (x11)	321.54053	2	146	149
			APAISVLHELIQQIFNLFTTK	1192.5554	2	74	94
33	Interferon gamma	P01579	KKRDDFEK (x7)	533.57104	2	110	117
			RDDFEK (x11)	405.42102	2	112	117
			KRSQMLFR (x4)	533.4254	2	153	160
			EESDR (x2)	635.4259	1	61	65
34	Tumor necrosis factor	P01375	DLSLISPLAQAVR (x2)	692.28796	2	66	78
			ANALLANGVELR (x2)	620.36035	2	109	120
			DVELAEEALPKK (x3)	671.00305	2	9	20
			TGGPQGSR	379.90448	2	21	28
35	Growth-regulated oncogene alpha protein	P09341	IIEKMLNSDKSN (x4)	696.01025	2	96	107
			ACLNPASPIVKK (x3)	620.36035	2	84	95
			RAAGASVATELR	601.51807	2	31	42
36	Interleukin-1 alpha	P01583	VLKK (x10)	488.16937	1	80	83
			LTFKESMVVVATNGKVLK	982.3987	2	65	82
			IIKYEFILNDALNQSIIR	1082.3301	2	129	146
37	Interleukin-1 beta	P01584	ISDHHYSK (x13)	493.63342	2	52	59
			IPVALGLK (x5)	405.4679	2	172	179
			KKMEK (x7)	664.3721	1	209	213
			FVFNKIEINNK	683.4104	2	215	225
38	Interleukin-2	P60568	ATELK (x7)	280.87842	2	70	74
			MYR (x9)	234.95697	2	1	3
39	Interleukin-12 subunit alpha	P29459	MCPAR (x13)	576.2787	1	1	5
			IKLCILLHAFR (x4)	664.2721	2	193	203
			TKIK (x10)	488.36182	1	191	194
			LLMDPKR (x2)	437.09894	2	145	151

Table S1. *Cont.*

#	Description	Accession n°	Sequence	m/z	z	Start	End
40	Interleukin-12 subunit beta	P29460	DQKEPKNK (x10)	493.63342	2	119	126
			REKK (x10)	280.6034	2	283	286
			AQDR (x11)	488.32056	1	310	313
			DIIKPDPPK (x3)	511.52173	2	231	239
			EKKDRVFTDKTSATVICRK (x2)	742.4414	3	284	302
41	Interleukin-15	P40933	TEANWVNVISDLK (x7)	744.332	2	46	58
			VTAMK	548.695	1	85	89
			ECEELEEK (x3)	504.0325	2	135	142
42	Hemoglobin subunit beta	P68871	AHGK (x3)	411.4612	1	63	66
			SAVTALWGK (x15)	466.0435	2	10	18
			VLGAFSDGLAHLDNLK	834.9495	2	68	83
43	Serum Albumin	P02768	LSQR	252.62006	2	243	246
			AACLLPKLDELR (x3)	671.00305	2	199	210
			TYETTLEK (x11)	492.8766	2	376	383
			LDELRDEGK (x12)	537.67285	2	206	214
			NLGKVGSKCCKHPEAK	849.97754	2	453	468
			AFKAWAVARLSQR	752.3341	2	234	246
44	Lysozyme C	P61626	TLKRLGMDGYR	437.21942	3	29	39
			VFER (x11)	275.7279	2	20	23
			CQNR (x2)	520.2289	1	134	137
			DPQGIRAWVAWRNRCQNR (x2)	742.4414	3	120	137
			CELARTLK	467.47418	2	24	31

Table S2. List of m/z SELDi-MS signatures corresponding to theoretical Mr data of proteins identified with LC-MS/MS.

SELDI-MS (m/z)	LC-MS/MS (theoretical Mr)	Protein
66032	66039	Keratin, type II cytoskeletal 1
62384	62378	Keratin, type II cytoskeletal 5
62061	62064	Keratin, type I cytoskeletal 9
51568	51562	Keratin, type I cytoskeletal 14
51906	51911	Keratin, type I cytoskeletal 26
26245	26242	Pulmonary surfactant-associated protein A1
26187	26182	Pulmonary surfactant-associated protein A2
22586	22580	Histone H1.5
46728	46737	Alpha-1-antitrypsin
10833	10835	Calgranulin A
13245	13242	Calgranulin B
21311	21307	Erythropoietin
39009	39000	Protein AMBP
8562	8565	Ubiquitin
11001	11006	Cystatin
71963	71957	Kininogen 1
11020	11025	MCP-1
21722	21725	Interferon alpha-1/13
19354	19348	Interferon gamma

Table S2. *Cont.*

SELDI-MS (m/z)	LC-MS/MS (theoretical Mr)	Protein
25647	25644	Tumor necrosis factor
11298	11301	Growth-regulated oncogene alpha protein
30743	30748	Interleukin-1 beta
17636	17628	Interleukin-2
24879	24874	Interleukin-12 subunit alpha
18078	18086	Interleukin-15
16601	15998	Hemoglobin subunit beta
16533	16537	Lysozyme C

© 2012 by the authors; licensee MDPI, Basel, Switzerland. This article is an open access article distributed under the terms and conditions of the Creative Commons Attribution license (http://creativecommons.org/licenses/by/3.0/).
